# Quantitative estimation of optical properties in bilayer media within the subdiffusive regime using tilted fiber-optic probe diffuse reflectance spectroscopy, part 2: probe design, realization, and experimental validation

**DOI:** 10.1117/1.JBO.29.10.105002

**Published:** 2024-10-29

**Authors:** Philippe De Tillieux, Maxime Baillot, Pierre Marquet

**Affiliations:** a Cervo Brain Research Centre, Québec, Canada; b Université Laval, Department of Physics, Physical Engineering, and Optics, Québec, Canada; c Université Laval, Department of Psychiatry and Neurosciences, Québec, Canada; d Centre for Optics, Photonics and Lasers, Québec, Canada; e Joint International Research Unit, Université Laval, Centre for Psychiatric Neuroscience, Department of Psychiatry, Lausanne University Hospital, University of Lausanne, Prilly, Switzerland

**Keywords:** diffuse reflectance, bilayer, subdiffusive, intrinsic optical properties, fiber optic probe, Monte Carlo simulation

## Abstract

**Significance:**

Tissues like skin have a layered structure where each layer's optical properties vary significantly. However, traditional diffuse reflectance spectroscopy assumes a homogeneous medium, often leading to estimations that reflects the properties of neither layer. There's a clear need for probes that can precisely measure the optical properties of layered tissues.

**Aim:**

This paper aims to design a diffuse reflectance probe capable of accurately estimating the optical properties of bilayer tissues in the subdiffusive regime.

**Approach:**

Using Monte Carlo simulations, we evaluated key geometric factors—fiber placement, tilt angle, diameter, and numerical aperture—on optical property estimation, following the methodology in Part I. A robust design is proposed that balances accurate intrinsic optical property (IOP) calculations with practical experimental constraints.

**Results:**

The designed probe, featuring eight illumination and eight detection fibers with varying spacings and tilt angles. The estimation error of the IOP calculation for bilayer phantoms is less than 20% for top layers with thicknesses between 0.2 and 1.0 mm.

**Conclusion:**

Building on the approach from Part I and using a precise calibration, the probe effectively quantified and distinguished the IOPs of bilayer samples, particularly those relevant to early skin pathology detection and characterization.

## 1 Introduction

Spatially resolved diffuse reflectance spectroscopy (srDRS) may be used as an optical biopsy tool for diagnostic applications, for example, to identify cancerous tissue.^1^^–^^3^ Practically, a specific tissue is characterized by a couple of optical coefficients, namely the absorption ($\mu_{a}$) and the reduced scattering coefficient ($\mu_{s}$) in a diffusive regime, and accompanied by the γ parameter in a subdiffusive regime. However, in tissues with a layered structure such as the skin, it is useful to be able to characterize each of these layers, which could have very different histoarchitectures, by obtaining their different intrinsic optical properties (IOPs). In addition, assuming a homogeneous medium while probing a layered medium may result in an estimate of the IOP that does not reflect that of either layer.^4^ To properly estimate the properties of a bilayer medium, two models exist in the literature: a sequential^5^^,^^6^ and a simultaneous model.^7^^,^^8^ The sequential model is carried out in two steps. In the first step, a probe geometry that minimizes the sampled depth, typically with fibers at shorter source-detector separations (SDS), is chosen to restrict the sampled volume within the upper layer. This means that the homogeneity assumption becomes valid and the IOPs of the upper layer can be estimated in the usual manner. Then, to estimate the properties of the deep layer, fibers placed at larger SDS are used to probe deeper into the tissue. The IOPs estimated in the first step are incorporated into the model, so the unknown parameters become the position of the interface and the IOPs of the deep layer. The benefit of the sequential model is that the complex bilayer problem is decoupled into two simpler problems. The main limitation is that the collected photons in the first step must have paths that are mostly confined to the upper layer. In Part 1 of this paper, it was shown, in particular with a probe with inclined fibers, that it is possible to correctly determine in a subdiffusive regime the optical coefficients of a biological tissue layer with a thickness as thin as ∼0.3  mm. This thickness may be too large for certain applications, particularly in dermatology, where the thickness of the upper layer, such as the epidermis or melanoma in its early stages, can be around 0.1 mm. In that case, a simultaneous model is necessary because it does not suffer from the same limitation. Indeed, there is no need to restrict the collected photons within the upper layer, as the photons are free to interact with both layers since their IOPs are evaluated simultaneously. In this second part of the paper, we propose the design of a probe with a geometry conducive to robust calculations of IOPs in the subdiffusive regime in the case of a bilayer medium. The limits of the numerical model presented in Part 1 of this paper are considered in the design. To achieve this design and validate it experimentally, several steps have been carried out. First, a numerical approach based on Monte Carlo (MC) simulations is presented to study the effects of probe geometry on the robustness and reliability of IOP calculations for a monolayer diffusive medium. Based on this numerical study, a probe geometry is proposed for calculating the six IOPs as well as the depth of the interface in a bilayer diffusive medium. The ability of this probe geometry to estimate the IOPs and depth of a bilayer diffusive medium is evaluated using MC simulations. Given the convincing numerical results, a custom-built probe with the proposed geometry is built and integrated into a typical DRS setup. Then, for validation purposes, experimental measurements performed on bilayer phantoms with known IOPs, which include an important calibration step are presented. Since one of the goals of this work is to develop a reliable approach for the detection and characterization of early epidermal lesions such as melanoma, we will often refer to the latter regarding typical size and characteristic depth of the volume of interest.

## 2 Numerical Study of the Probe Geometry on IOP Estimation

**Fig. 1:**
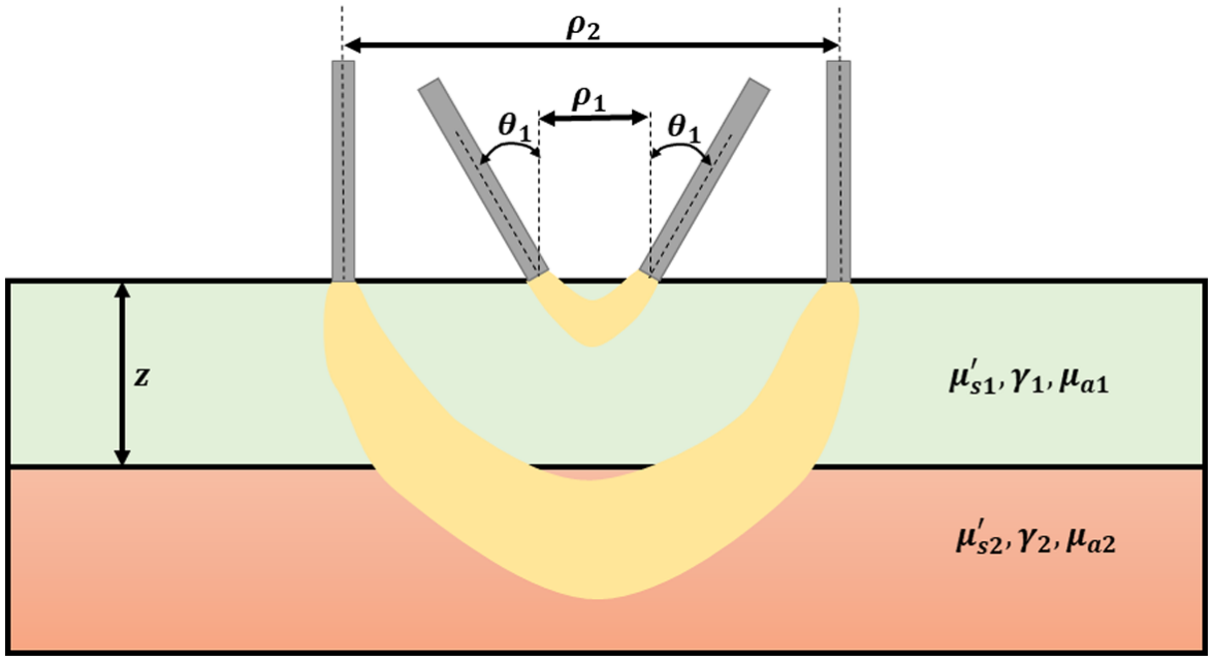
Schematic of the DRS setup for two fiber combinations for a bilayer medium.

In srDRS, a typical setup uses an illumination fiber and several detection fibers to measure the backscattered reflectance spectrum as a function of SDS. When working in the subdiffusive regime with a model free of spectral priors, there are three unknowns to estimate at each discretized wavelength $\left(\mu_{a},\mu_{s}^{\prime },\gamma \right)$. The inverse problem of estimating the IOPs from the measured backscattered signal is nonlinear.^9^ For a bilayer medium, the number of unknowns increases to seven: the IOPs of each layer and the position of the interface, which is represented in Fig. 1. This higher number of unknowns increases the complexity of the inverse problem and suggests that the number of independent measurements should be increased to obtain a similar well-posed problem. Most research published on the impact of probe geometry focuses on the sampled depth rather than its effect on IOP estimation.^10^^,^^11^ In this section, the effect of fiber placement, tilt angle, number of measurements, fiber diameter, and numerical aperture on the IOP estimation are analyzed. Two approaches are used to conduct this study: the first is based on studying the shape of the cost function as a function of probe geometry, and the second is based on the mean estimation error of the inverse solver using synthetic noisy data.

### 2.1 Fiber Placement

For the second-order approximation of the phase function to hold, the minimal SDS must respect the criterion presented in Part 1 of this paper, i.e., that the collected photons for each fiber combination scatter enough times for the effect of the moments of order higher than 2 to be negligible. For varying SDS values, the cost function F is calculated from the relative difference between synthetic noisy data and a large look-up table (LUT). The synthetic data is generated by computing the reflectance of a homogenous medium for a triplet of optical coefficients typically representative of a biological tissue^12^ ($\mu_{s}^{\prime }=2\;{\mathrm{mm}}^{-1}$, γ=1.5, $\mu_{a}=0.2\;{\mathrm{mm}}^{-1}$, labeled case #1) using an MC simulation to which 10% white Gaussian noise is added. For all simulations of biological tissue, the refractive index is set to 1.43. The LUT is generated to cover a wide range of possible values for biological tissues: $\mu_{s}^{\prime }\in [0.5,3]\;{\mathrm{mm}}^{-1}$, γ=[1.0,2.0], $\mu_{a}\in [0,1.0]\;{\mathrm{mm}}^{-1}$ with 40 discretized points along each axis.

**Fig. 2:**
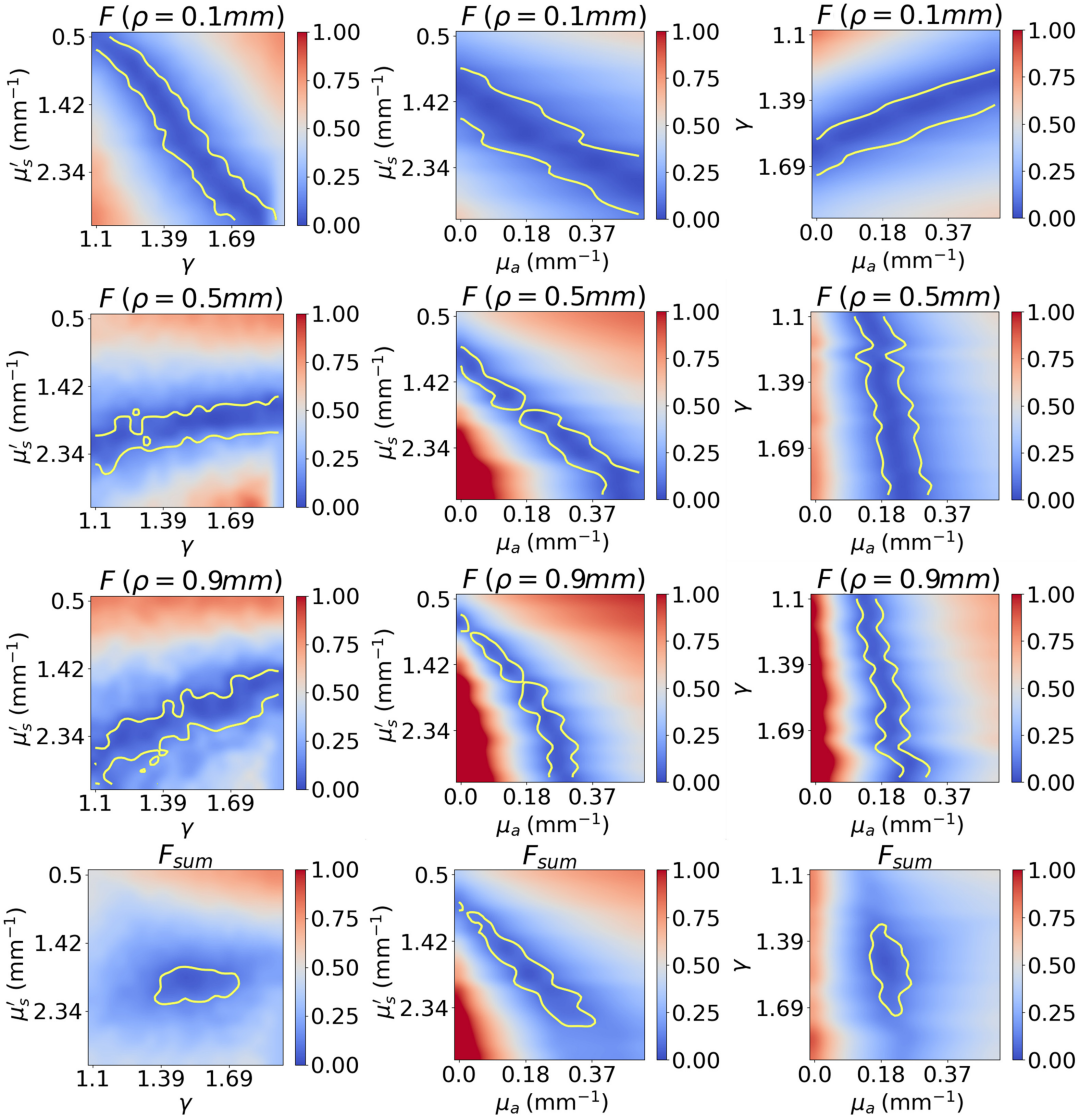
Cost function in the $\left(\mu_{s}^{\prime },\gamma \right)$, $\left(\mu_{s}^{\prime },\mu_{a}\right)$ and ($\left(\gamma ,\mu_{a}\right)$ planes for fibers at SDS of 0.1, 0.5, and 0.9 mm and for the average of the three fibers. The yellow contour line delimits the region where the value of the cost function is less than 10%.

The 3D visualization of the cost function is strenuous, so it may be preferable to visualize each of the 2D planes, where the value of the third axis corresponds to the one used for generating the synthetic reflectance. Figure 2 shows the cost function in each of the 2D planes for fibers at SDS of 0.1, 0.5, and 0.9 mm and for the sum of these three fibers. The yellow contour line delimits the minimum region where the cost function is less than 10%. We notice that in each 2D plane, the surface of the minimum is elongated along a certain direction and that this direction rotates as a function of SDS, which is particularly noticeable for SDS of 0.1 and 0.5 mm. For larger SDS, the main difference seems to be that the surface of the minimum becomes thinner along the $\mu_{a}$ axis. By averaging the individual cost functions of the three fibers, the minimum region becomes more compact than any individual fiber allowing thus more accurate calculation of optical coefficients. It thus appears clearly that it is essential to use fibers at multiple SDS to obtain a compact cost function. Further, it seems that the fibers at small SDS are of particular importance for estimating the scattering coefficients because the minimum region is more orthogonal than for fibers at larger SDS, which seem to mainly contribute to a more accurate estimation of the absorption coefficient.

### 2.2 Tilt Angles

**Fig. 3:**
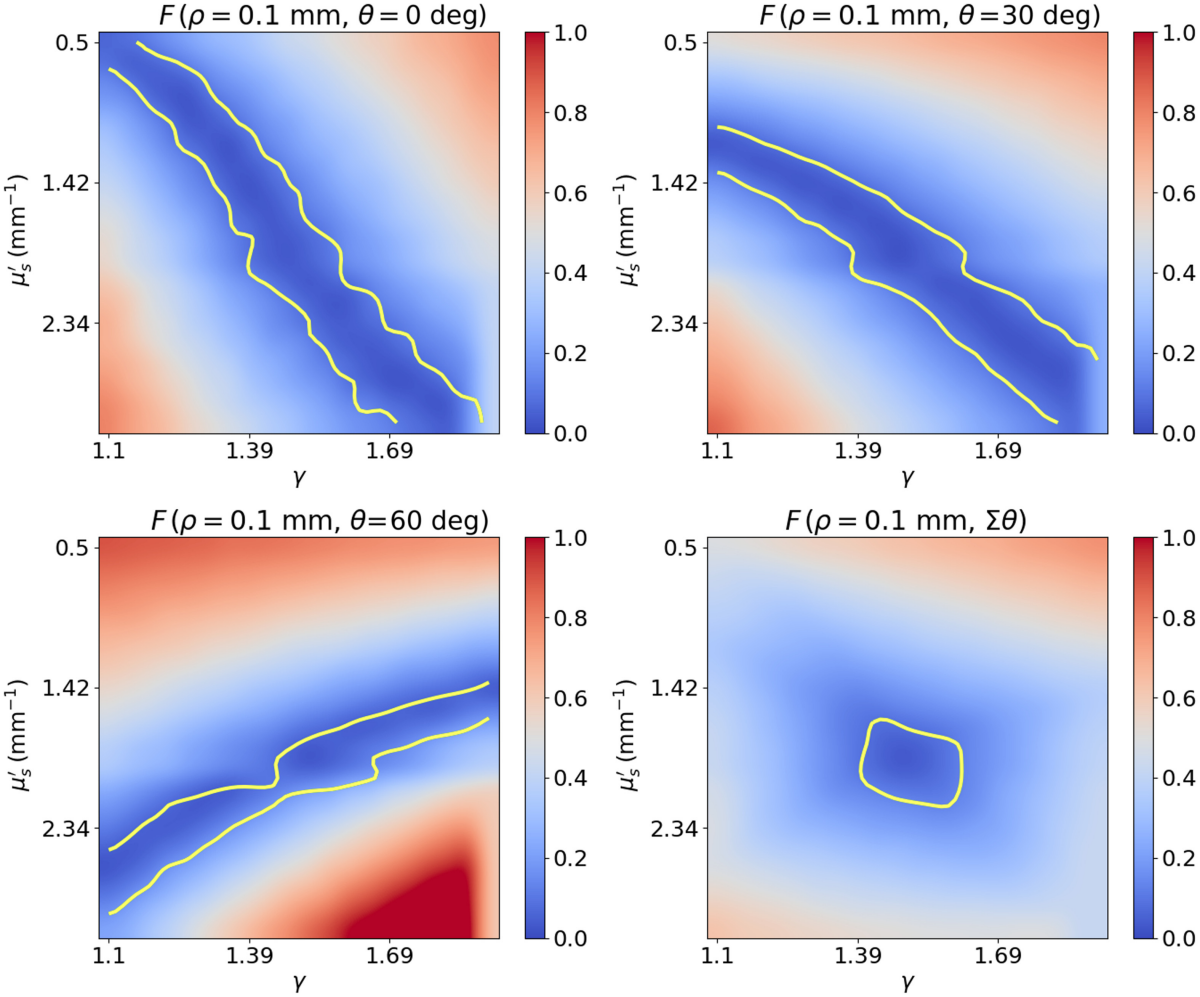
Cost function in the $\left(\mu_{s}^{\prime },\gamma \right)$ plane for fibers at an SDS of 0.1 mm for tilt angles of 0 deg, 30 deg, and 60 deg and for the average of the three fibers. The yellow contour line delimits the region where the value of the cost function is less than 10%.

Tilted fibers in srDRS have been used in several publications with the goal of reducing the sampled depth of the collected photons,^13^^–^^15^ but their impact on IOP estimation has not been thoroughly studied. Figure 3 shows the cost function for a fiber at an SDS of 0.1 mm and with tilt angles of 0 deg, 30 deg, and 60 deg and for the average of these fibers. It can be noticed that the effect of varying the tilt angle is similar to that of varying the SDS. The minimum region of the cost function rotates in the $\left(\mu_{s}^{\prime },\gamma \right)$ plane and the cost function averaged over the three tilt angles becomes more convex and compact than for any of the individual combinations. This is because fibers at different tilt angles collect photons that sample the scattering phase function differently. For example, for perpendicular fibers, a backscattering event is generally required for the photons to reach the detection fiber, whereas for higher tilt angles, the collected photons are scattered in a more forward direction. Further, lower tilt angles are associated with longer path lengths, which provides different information about the absorption coefficient than higher tilt angles, which correspond to shorter path lengths. Figure 14 in the Appendix shows a second example, this time for fibers at an SDS of 1.9 mm in the $\left(\mu_{s}^{\prime },\mu_{a}\right)$ plane. Similarly, we notice a change in the shape of the minimum region for different tilt angles, which results in a more local and better-defined total cost function.

### 2.3 Number of Measurements

**Fig. 4:**
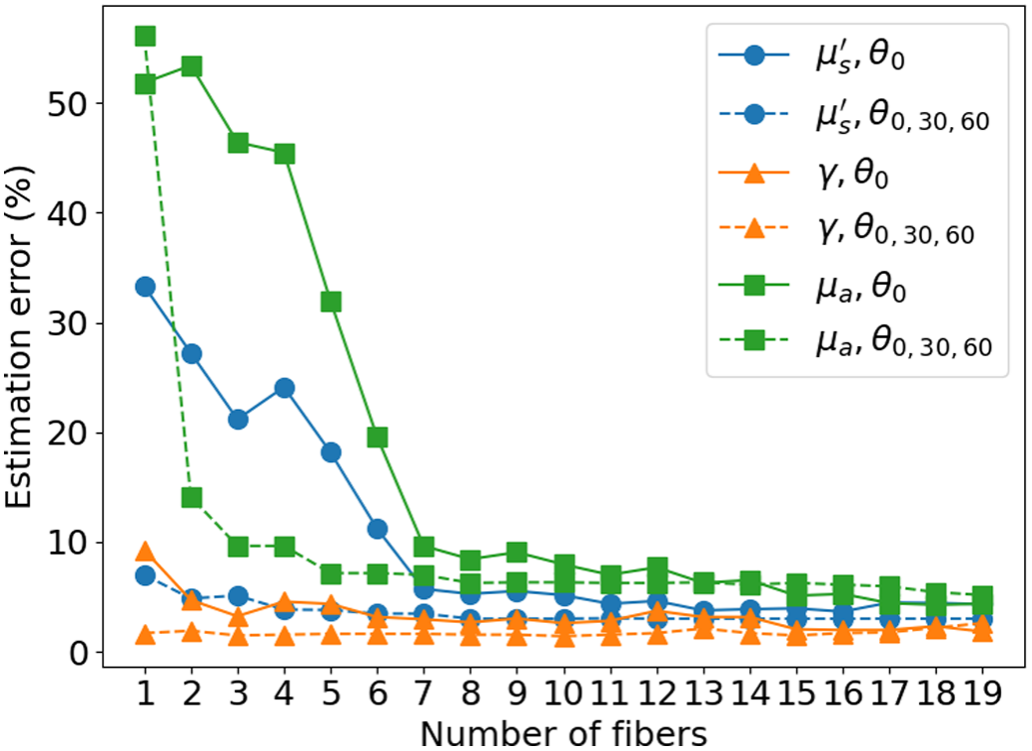
Estimation error as a function of the number of detection fibers while using evenly distributed SDS between 0.1 and 2.0 mm with only perpendicular fibers and with fibers at 0 deg, 30 deg, and 60 deg.

Each measurement in srDRS contains a certain noise level. Assuming an identically distributed noise for each measurement, the higher the number of measurements, the better the reflectance curve is defined. The estimation error is thus expected to decrease as a function of the square root of the number of measurements. To visualize this effect, synthetic data is generated by adding 10% white Gaussian noise to the MC simulation results and this data is fed to the inverse solver with varying geometries of the optical setup. The estimation error as a function of the number of detection fibers is represented in Fig. 4. The minimum and maximum SDS are set to 0.1 and 2.0 mm, and the number of detection fibers evenly distributed between those two SDS is gradually increased. The solid lines represent the estimation error obtained solely with perpendicular fibers, whereas the dashed lines represent the estimation error obtained using three fiber arrays at the same SDS but with tilt angles of 0 deg, 30 deg, and 60 deg. The property most sensitive to the number of measurements appears to be the absorption coefficient. The estimation error initially decreases very rapidly with additional fibers, but the improvement becomes slower as the number of measurements increases, which corresponds to the expected behavior. Further, we see that the convergence of the estimation error is drastically improved using a variety of tilt angles. This is because, by varying the fiber tilt angle, a wider distribution of path lengths and a more varied sampling of the scattering phase function is measured. This richer information contained in the collected photons about the scattering medium contributes to a more compact cost function, which translates to a more robust IOP estimation. It is worth noting that we here compare one perpendicular fiber to three detection fibers (at different tilt angles) at the same SDS. The goal of this figure is not to compare the improvement brought to the robustness of the inverse solver by varying the SDS to the improvement brought by varying the fiber tilt angle, because this improvement depends on a myriad of parameters (e.g., tissue IOP and parameter ranges considered). The goal is rather to showcase the improvement brought using varied tilt angles compared to using only one, as this brings additional information about the sampled medium, which contributes to a more convex and better-defined cost function and an improved robustness of the inverse solver.

### 2.4 Diameter and Numerical Aperture

**Fig. 5:**
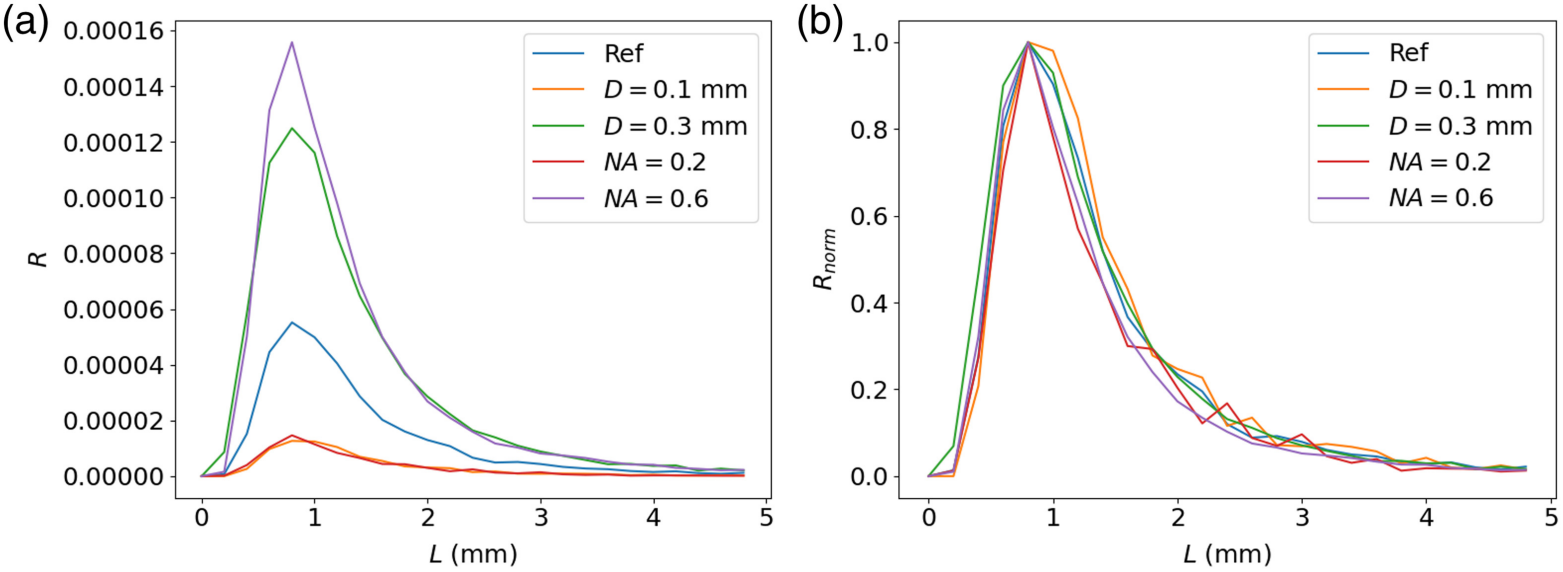
(a) Absolute and (b) normalized reflectance as a function of photon path length for different fiber diameters and numerical apertures. The reference value is for D=0.2  mm and NA=0.4.

Figure 5(a) shows the path length distribution of the collected photons for perpendicular fibers at an SDS of 0.4 mm. The reference value is for a diameter of 0.2 mm and a numerical aperture of 0.4. We see that the fiber diameter and numerical aperture greatly affect the number of collected photons. However, when normalizing the histograms so that their maximum bin content is unitary, it can be observed in Fig. 5(b) that fiber diameter and numerical aperture do not significantly affect the collected photon’s path length distributions.

## 3 Proposed Geometry and Numerical Characterization

### 3.1 Probe for Melanoma Detection

To design an srDRS probe that can robustly estimate the IOPs of bilayer diffusive media, several aspects must be considered, which are summarized in Table 1. The probe performances to optimize are the precision, i.e., the minimization of the estimation error, and the robustness, i.e., the capacity to obtain a precise result even in the presence of noisy data. We propose to optimize IOP estimation by adjusting fiber placement, tilt angle, number of measurements, fiber diameter, and numerical aperture. These geometrical parameters are constrained by certain limits. First, the minimal SDS must satisfy the criterion presented in Part 1 of this paper (F>0.1). If this criterion is not met, the phase function moments of order greater than 2, which are not accounted for in the inverse solver, will significantly influence the reflectance signal. This may lead to erroneous IOP estimation. Second, the transverse sampled surface should remain within the region of interest. In the case of melanoma, a reasonable objective is to restrict the collected photons within a circular region of 3 mm diameter. This size is chosen because 95% of melanomas have a diameter greater than this value at the time of diagnosis.^16^ Third, the depth sampled by light should be of the same order of magnitude as the volume of interest. For melanomas, it is of particular interest to be able to identify them at their first development stages because, at that moment, they are harder to distinguish from melanocytic nevi. A Breslow depth, i.e., the thickness of the melanomas within the skin, of 0.75 mm is used clinically to assess the severity of melanomas, as survival rates greatly reduce for melanoma thickness larger than this value.^17^ Fourth, the optical design should be simplified as much as possible to decrease the cost and the burden of probe manufacturing.

**Table 1:** Parameters of probe performance, geometric parameters, and experimental and numerical constraints considered for the novel DRS probe design.

Probe performance	Geometric parameters	Constraints
• Precision	• Fiber placement	• Limits of second-order approximation
• Robustness	• Tilt angle	• Transverse sampled area
	• Number of measurements	• Sampled depth
	• Diameter, numerical aperture	• Optical design complexity

**Fig. 6:**
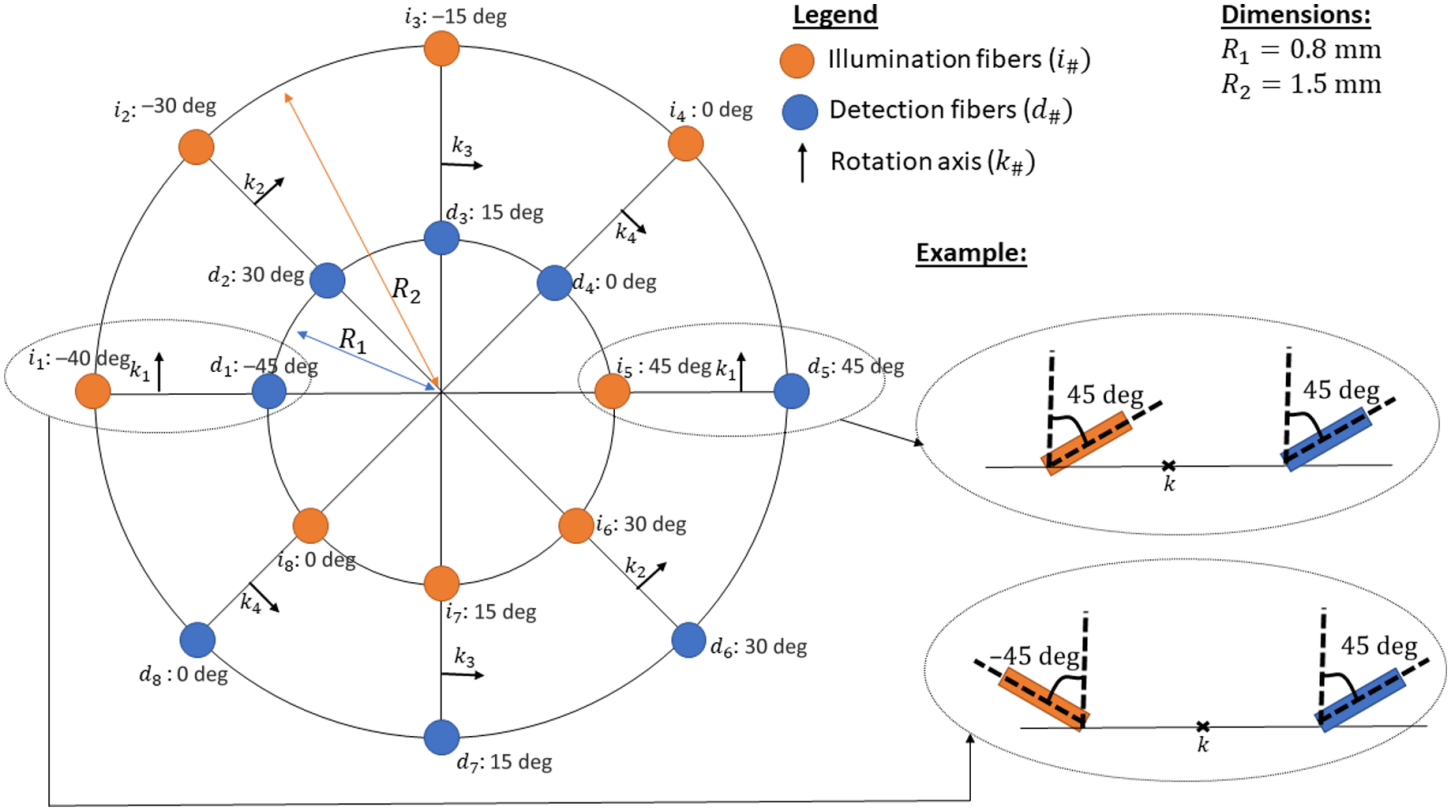
Schematic of the transverse plane for the probe geometry and fiber identification.

The effect of each geometrical parameter has been shown in Figs. 2–5. It seems desirable to use the smallest possible $\rho_{\min}$ value that satisfies the condition for the second-order approximation of the phase function and the largest possible $\rho_{\max}$ value that allows the sampled surface to remain within the region of interest. The number of measurements should be increased as much as possible within the constraints of the experimental probe realization, and the fiber tilt angles should be varied as much as possible to maximize the information about the sampled volume contained in the collected photons. Figure 6 shows the proposed design geometry, using eight illumination fibers and eight detection fibers distributed around circles of 0.8 and 1.5 mm radius. All of the fibers on the larger circle are tilted inward to keep the sampled surface within the surface of the melanoma. The rotation angles are given in reference to the rotation axes k indicated along for each set of fibers on the circle’s radii. The tilt angles and the fiber placement have been distributed to obtain fiber combinations that sample distances close to the theoretical limit of $\rho_{\min}$ as well as distances at the maximal SDS possible for the considered application. The remaining fibers have been evenly distributed to provide a set of collected photons with varied distributions of path lengths and sampling of the scattering phase function. The reason for using a homogeneous distribution of the SDS and tilt angles is based on a study of fiber placement within homogeneous diffusive media,^18^ where the ideal distribution obtained through stochastic optimization was shown to be nearly identical to a homogeneous distribution. The precise number of illumination and detection fibers is chosen for three reasons. First, there is a practical limit to the number of fibers that can be inserted within the 3-mm diameter of the probe tip. There are practical constraints on the wall thickness between the holes into which the fibers are inserted because they would otherwise collapse into one larger hole. Second, an equal number of illumination and detection fibers maximizes the number of different fiber combinations. Third, there are readily available 1×8 optical switches that can sequentially alternate the signal from one fiber to eight other fibers. A relatively small fiber diameter of 0.1 mm is chosen to allow the fibers to be inserted within the 3-mm diameter of the probe’s surface. The numerical aperture of the fiber is chosen to be 0.22 to match the numerical aperture of the rest of the optical setup, e.g., the source, spectrometer, and optical switch. The tilted fibers are beveled at an angle equal to their fiber tilt angle so that the fiber’s surface is flush with the probe tip. This beveling of the fibers modifies the effective tilt angle of the photons as they exit the illumination fibers or enter the detection fibers. Indeed, the effective tilt angle is increased by a value of $\gamma_{B}=\sin^{-1}(n_{f}\;\sin(\beta ))-\beta$, where β is the tilt angle.^19^ For example, for a tilt angle of 30 deg, the beveling adds another 10 deg to the effective tilt angle.

To evaluate the probe’s capacity to estimate the IOPs of bilayer media, namely the reduced scattering coefficients ($\mu_{s}^{\prime }$), the subdiffusive parameter (γ), and the absorption coefficient ($\mu_{a}$) of both layers, as well as the position of the interface between both layers (z), a large 7D LUT is generated using MC simulations for the probe geometry shown in Fig. 6. The number of independent MC simulations required to generate the LUT is on the order of $n_{x}^{5}$, where $n_{x}$ is the number of discretized values along each IOP axis. This is because new simulations are not required for different $\mu_{a}$ values since the path lengths are stored in the memory and the weight of each collected photon packet is weighed according to the Beer-Lambert equation. Due to the fiber tilt, a radial symmetry cannot be exploited to accelerate the convergence of the MC simulation.^20^ The number of launched photons is set to $N=10^{8}$ to maintain a mean uncertainty of 4% across all combinations of illumination and detection fibers as presented in Sec. 4.2.1. The enormous computational cost associated with computing $10^{5}$ independent MC simulations with $10^{8}$ photons each is alleviated using two strategies. The first strategy is to use a GPU-based MC simulation program^21^ that can drastically reduce the computation time of the individual simulations. Indeed, MC simulations are often described as embarrassingly parallel,^22^ which makes them perfect candidates for GPU computations. The second strategy is to distribute the independent simulations across several computation nodes on the Digital Research Alliance of Canada servers.^23^ Using these two strategies, the computation time to simulate approximately one million independent MC simulations with $N=10^{8}$ photons each is reduced to five days. The grid ranges cover $10\;\mu_{s}^{\prime }$ values in $[0.25,3]\;{\mathrm{mm}}^{-1}$, 10 γ values in [1.0, 2.0], $20\;\mu_{a}$ values in $[0,0.2]\;{\mathrm{mm}}^{-1}$ for each layer, and 15 z values in [0.1,1.5]  mm. These values are chosen to cover a wide range of realistic IOPs.^12^

**Fig. 7:**
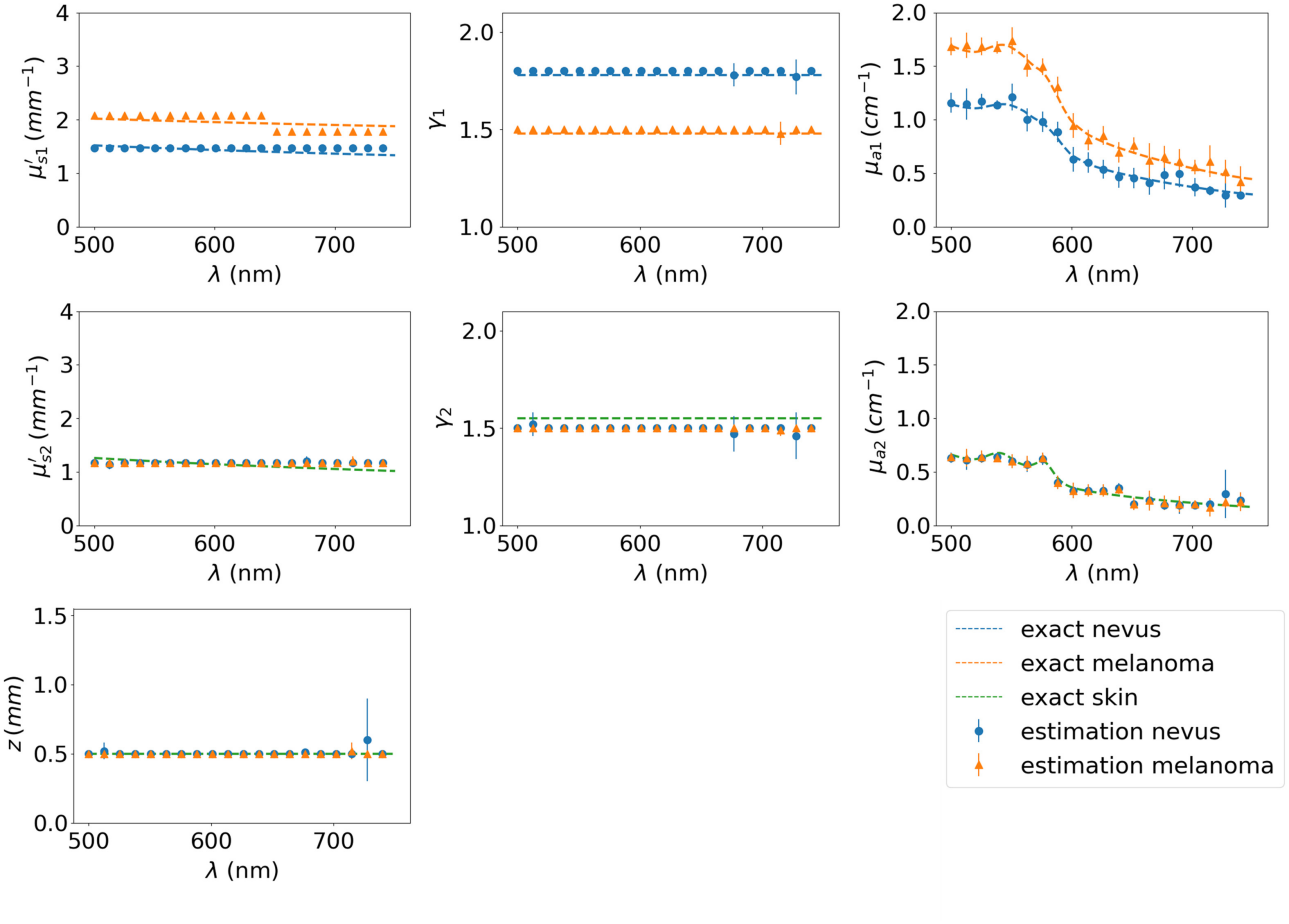
Spectral estimation of the IOPs for a nevus (blue circles) and a melanoma (orange triangles), as well as the exact IOP values (dashed lines) used to generate the synthetic data with 5% of added white Gaussian noise.

As an example of the numerical model’s capacity to estimate bilayer media IOPs, synthetic data with a 5% added white Gaussian noise are generated using the theoretical spectra of a melanocytic nevus in the skin and of melanoma in the skin,^24^ both with a thickness of 0.5 mm. Figure 7 shows the exact IOP spectra used to generate the synthetic data and the IOP estimated by the inverse solver. We see that all properties are correctly identified and that the melanocytic nevus can be distinguished from melanoma. The proposed probe design generates a fairly well-posed inverse problem that seems promising for discriminating melanoma from melanocytic nevi, but the accuracy of the estimation may be degraded for data with a lower signal-to-noise ratio (SNR), for a smaller thickness of the upper layer, or for a lower contrast value between both layers, which are described in Sec. 3.2.

### 3.2 Numerical Characterization of the Probe’s Limits

**Fig. 8:**
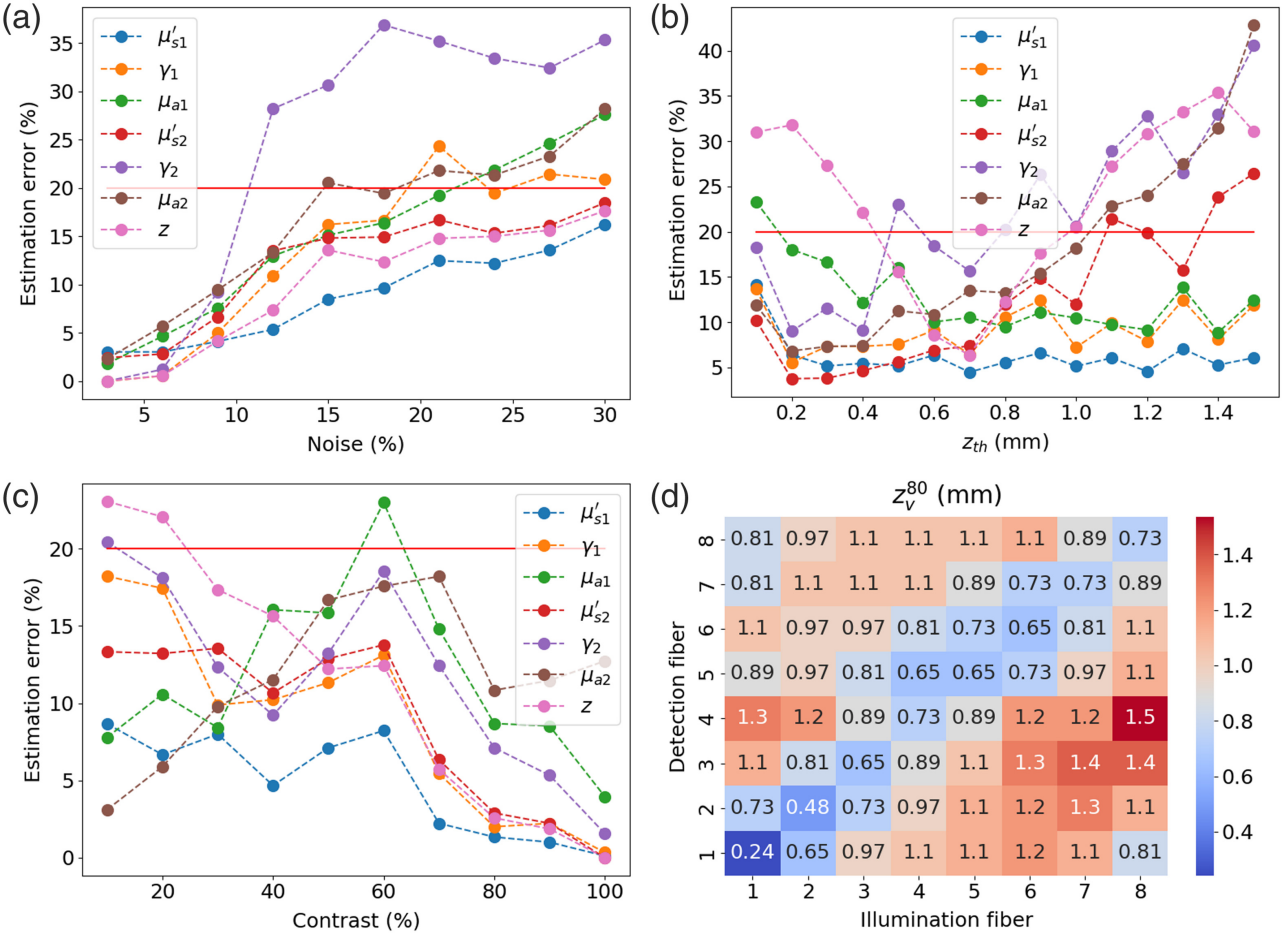
Estimation error as a function of (a) noise, (b) upper layer thickness, and (c) contrast using the proposed probe geometry with synthetic data. In panel (d) is the sampled depth ($z_{v}^{80}$) for each combination of illumination and detection fiber.

To analyze the numerical limits of the designed probe, synthetic data are generated for varying cases. The model is expected to behave similarly at each discretized wavelength, so only the optical properties at 540 nm typical of a skin melanoma, which in the present situation has a thickness of 0.7 mm ($\mu_{s1}^{\prime }=2.0\;{\mathrm{mm}}^{-1}$, $\gamma_{1}=1.78$, $\mu_{a1}=0.16\;{\mathrm{mm}}^{-1}$, $\mu_{s2}=1.1\;{\mathrm{mm}}^{-1}$, $\gamma_{2}=1.55$, $\mu_{a2}=0.06\;{\mathrm{mm}}^{-1}$, z=0.7  mm) is used to perform this analysis. In Fig. 8(a), a progressively higher noise value is added to the synthetic data, and the estimation error for each IOP is evaluated accordingly. The process of generating synthetic data and estimating the IOPs is repeated 20 times to obtain a statistically significant result. We see that the estimation error is approximately linear with the level of added noise, except for the estimation error of $\gamma_{2}$, which drastically increases at around 13% of added noise. This large error in $\gamma_{2}$ appears to be due to the coarse discretization of the LUT, as only 10 values are available along each of the LUT axes. Figure 8(b) shows the mean estimation error after 20 trials as a function of the theoretical upper layer thickness ($z_{th}$, i.e., melanoma thickness) with 10% white Gaussian noise added. We see that, for small $z_{th}$ values, the estimation error is highest for $\mu_{a1}$ and z. This is because the collected photons have a short path length within the upper layer, so the backscattered signal is less sensitive to those properties. As zth increases, the error in the properties of the upper layer decreases, and the error in the properties of the deep layer increases. This is because the thicker the upper layer is, the lower the proportion of backscattered photons that have reached the deep layer. In Fig. 8(c), the properties of both layers are set to the minimum simulated value of the LUT ($\mu_{s}^{\prime }=0.25\;{\mathrm{mm}}^{-1}$, γ=1.0, $\mu_{a}=0\;{\mathrm{mm}}^{-1}$), and the three properties of the deep layer are progressively increased to cover the full range of simulated values in the LUT. We see that as the contrast between both layers increases, i.e., the differences between their respective IOPs, the estimation error tends to decrease, with a clear drop in estimation error for contrast values larger than 60%. For a low contrast value, the most difficult property to estimate seems to be z. This is because the effect of the upper layer thickness on the backscattered signal is lesser than when the contrast is more important. We also note that the absorption coefficients’ estimation errors do not initially reduce with increased contrast, but they eventually do with a high enough contrast. This may be caused by the complex interaction between the absorption coefficients and the sampled volume, i.e., an increased absorption reduces the photon’s path length within the medium, leading to fewer interactions, which can reduce the information contained in the measured signal. Finally, Fig. 8(d) shows the sampled depth ($z_{v}^{80}$) presented in Part 1 of this paper for each combination of illumination and detection fibers. We see that the sampled depth is minimal for fibers at short SDS with high tilt angles, with a minimum at 0.24 mm for combination 1-1, and that few combinations probe at a depth of more than 1.1 mm, which explains why the estimation error in the IOPs of the deep layer increases rapidly for zth values greater than 1.1 mm in Fig. 8(b).

## 4 Experimental Validation

### 4.1 Optical Setup and Probe Realization

**Fig. 9:**
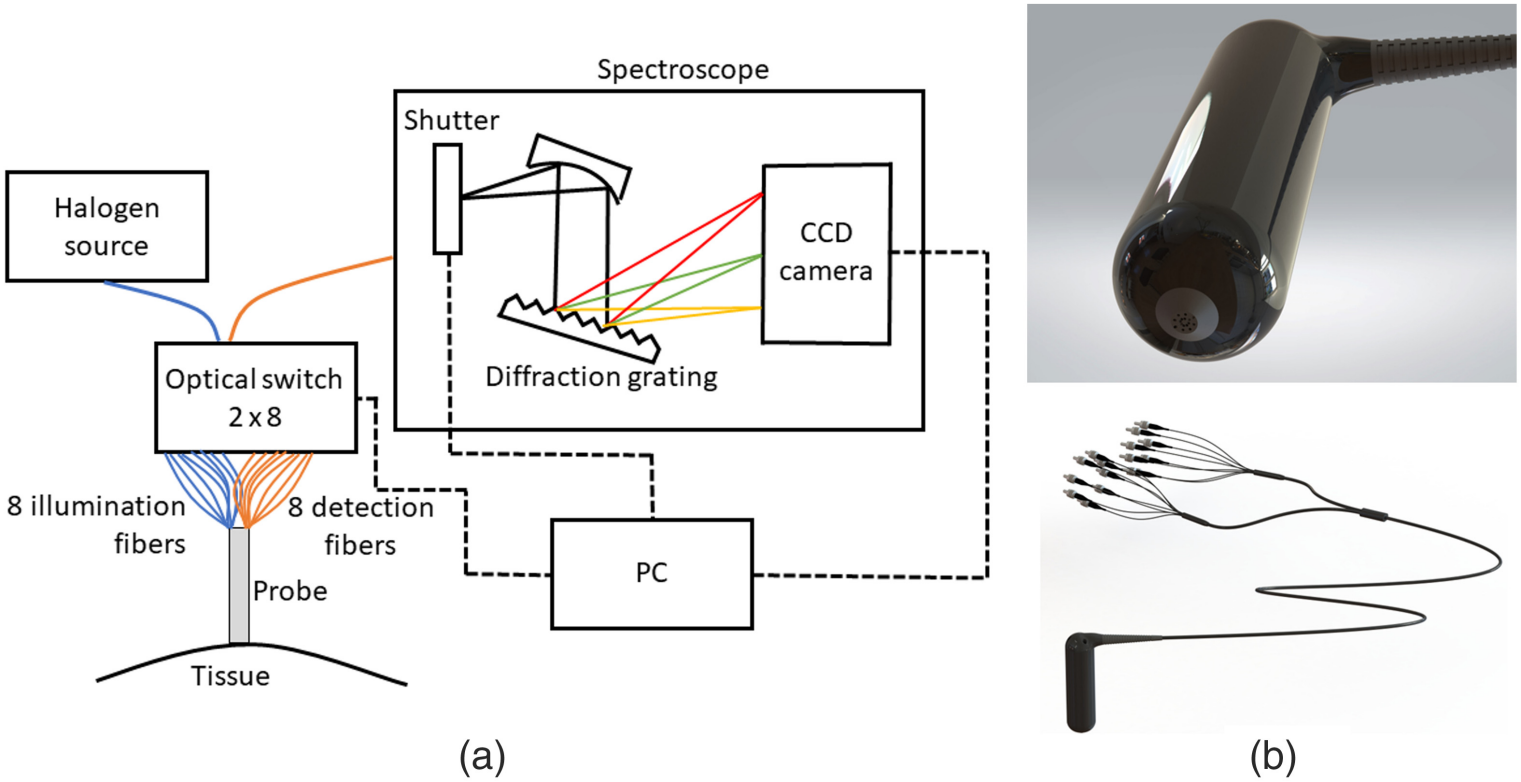
Probe realization: (a) Schematic of the optical setup integrating the custom-built probe. (b) Fiber tip with a 3-mm diameter and complete probe with eight illumination fibers and eight detection fibers.

A typical DRS setup is represented in Fig. 9(b). The light source used is a halogen lamp (HL-2000-HP from OceanInsight). It is selected because of its broad spectrum with a maximum between 500 and 750 nm; its intensity, which is high enough to generate a substantial backscattered signal without inducing hyperthermia in the tissue; its stability over time; and its common use in DRS setups.^25^^–^^27^ The optical switch is custom-built by the company Leoni to simultaneously and independently control two separate 1×8 switches to sequentially alternate the signal from each illumination and detection fiber. The chosen spectroscope (HR2000+ from OceanInsight) works between 200 and 1100 nm, has a spectral resolution of the order of 1 nm, an SRD of 250:1, and its integration time can be adjusted between 1 ms and 65 s. A laptop controls the optical switch and the spectroscope shutter and records the CCD camera’s signal. An integration sphere (FOIS-1 from OceanInsight) and optical phantoms (Biomimic Optical Phantoms by INO^28^) are used to calibrate the DRS probe. A solid phantom was preferred over a microbead solution because of its stability over time, its experimentally validated IOPs, and its ability to be manufactured in specific geometries, which is essential for forming bilayer media.^29^

The probe containing the 16 fibers is realized using 3D printing. The structure is protected by an outer layer that encompasses the fibers and allows easier manipulation, which can either be hand-held or placed into a holder. Because the holes into which the optical fibers slide must be slightly larger than the fibers to insert them without risking damage, there is a small uncertainty in the exact positioning of the fibers at the probe tip. Figure 15 in the Appendix shows the image of the probe tip acquired by optical microscopy. To evaluate each fiber’s exact position, image analysis is performed to obtain the exact center of each fiber and incorporate these values in the MC simulation.

### 4.2 Signal Processing

**Fig. 10:**
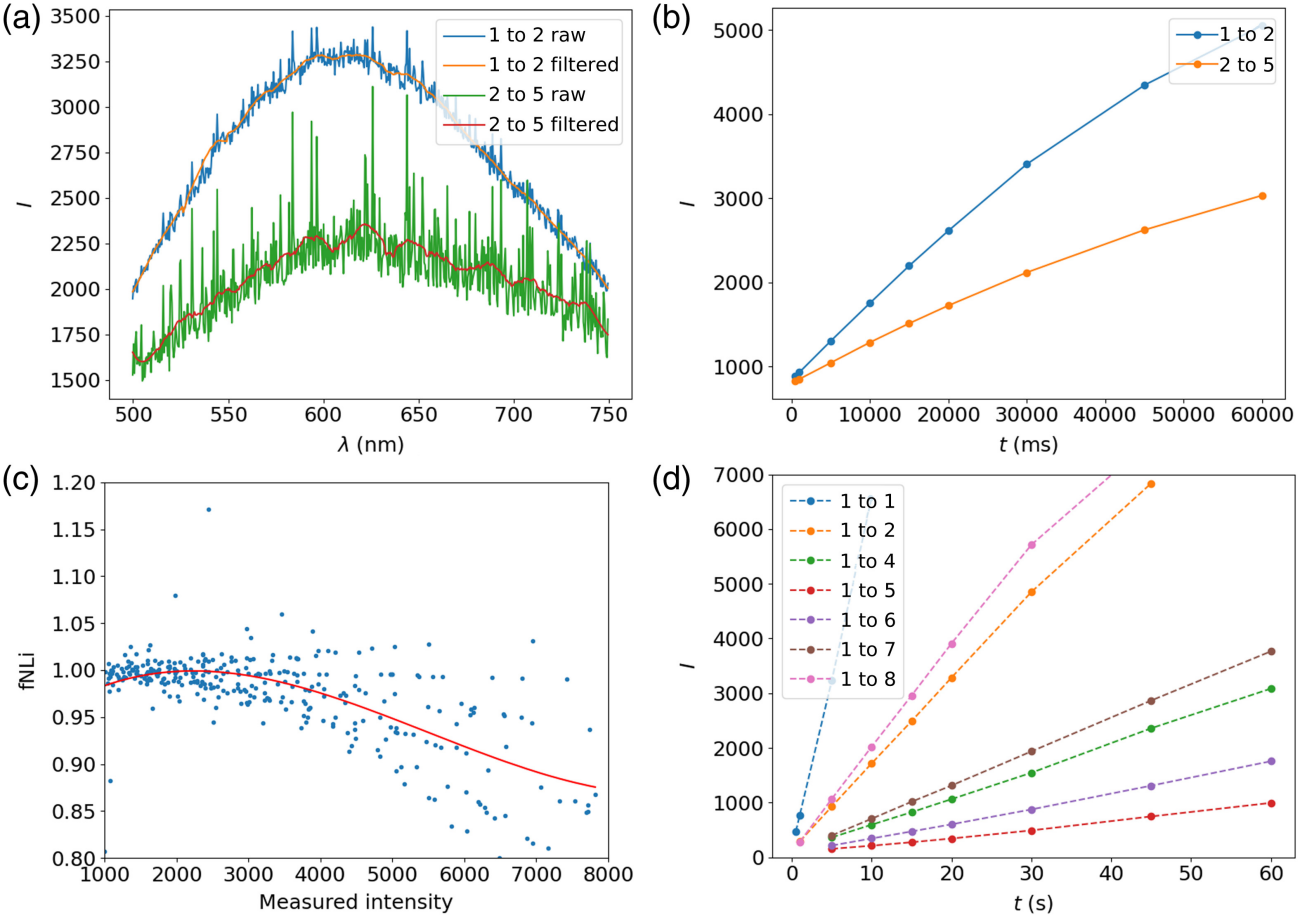
Steps of the signal processing. (a) Savitzky-Golay filter to filter out spectral noise. (b) Nonlinearity correction for long integration times by measuring the signal without the source. (c) Nonlinearity measurement of the detector where the points represent the ratio of the measured intensity over the expected intensity with a linear fit as a function of integration time. The total non-linearity factor is fitted by minimizing the RMSE using a third-degree polynomial. (d) Corrected signals after the signal processing algorithm is applied.

The signal measured by the spectrometer is degraded by several noise sources, namely:

• Thermal and shot noise that creates random high-frequency noise in the measured spectrum [Fig. 10(a)]. This noise is reduced by applying a smoothing filter, in this case, a Savitzky-Golay filter of order 3 with a window length of 21.
• A constant value ($I_{\mathrm{DC}}$) that is present even when the CCD integration time is set to zero. This bias is computed by performing a linear fit of the signal as a function of integration time [Fig. 10(b)].
• Ambient light ($I_{\mathrm{amb}}$) and dark current ($I_{\text{dark}}$), which increases the measured signal linearly as a function of time even when the halogen source is turned off. This component is also evaluated from the slope of linear fit in Fig. 10(b), which represents the summation of $I_{\mathrm{DC}}$, $I_{\mathrm{amb}}$, and $I_{\text{dark}}$ since it is measured signal with the source is turned off.
• Nonlinearity due to the high integration times that result from a temperature drift in the spectrometer.^30^ This component is corrected by computing the ratio of the measured intensity as a function of time with the source turned off in Fig. 10(b) over the values of the estimated linear fit. It is easily noticeable that acquisitions at higher exposition times no longer follow a linear distribution, which must be corrected by the non-linearity factor $f_{\mathrm{NL}}$.
• Nonlinearity due to the large variation in signal amplitude.^31^ To evaluate this component, the signal is acquired as a function of the integration time with the source turned on. The previously described noise sources are corrected for (spectral noise, bias, ambient light, and temporal nonlinearity), and a linear fit is performed for each fiber combination. The ratio of the measured value over the value of the linear fit for each integration time of each fiber combination is reported in Fig. 10(c). We see that, even though the data is considerably noisy, there is a clear tendency (represented by the adjusted third-degree polynomial in red) for the CCD to be less effective at higher intensities, which corresponds to the expected results.^31^

To summarize, the raw and pure signals are related by^30^

$$I_{\text{raw},\text{filt}}=I_{\mathrm{DC}}+f_{\mathrm{NL}}(I_{\text{dark}}(\lambda )+I_{\mathrm{amb}}(\lambda )+I(\lambda ))t$$ (1)

where $I_{\text{raw},\text{filt}}$ is the raw measurement smoothed with the Savitzky-Golay filter to reduce thermal and shot noise, and $f_{\mathrm{NL}}$ is the nonlinearity correction for the detector that accounts for both exposition time and light intensity. Figure 10(d) represents the signal collected by each detection fiber using illumination fiber 1 as a function of integration time after the signal processing algorithm is applied, which significantly improves the linearity measurements. Detection fiber 3 was damaged during the probe manufacturing, so it is excluded from the measurements.

#### 4.2.1 Calibration

The reflectance obtained from MC simulations corresponds to the backscattered signal that enters the detection fibers via the signal that exits the illumination fibers. However, the measured signal of an object of interest is not directly the reflectance of the object ($R_{\mathrm{obj}}$). It corresponds to the intensity of the signal at the CCD camera and is affected by each optical component, e.g., by the intensity of the source ($I_{\text{source}}$), the losses in the illumination and detection fibers ($L_{\mathrm{ill}},L_{\det}$), the losses in the optical switch ($L_{\text{switch}}$), and the efficiency of the spectrometer ($L_{\text{spectro}}$), and is thus given by

$$S_{\mathrm{obj}}(\lambda )=I_{\text{source}}(\lambda )L_{\mathrm{ill}}(\lambda )L_{\text{switch}}(\lambda )R_{\mathrm{obj}}(\lambda )L_{\det}(\lambda )L_{\text{switch}}(\lambda )L_{\text{spectro}}(\lambda ).$$ (2)

By replacing the object of interest with an integrating sphere, the path of the photons within the optical setup remains the same. The sphere’s theoretical reflectance is unitary and spectrally invariant, but because it contains an aperture in front of which the probe is placed, the intensity is decreased by a scalar value α referred to as the calibration coefficient, which is an unknown parameter that must be estimated. This implies that the experimental reflectance is given by

$$\alpha R_{\exp}(\lambda )=\frac{S_{\mathrm{obj}}}{S_{\mathrm{sph}}}.$$ (3)

To determine the calibration coefficient α for each fiber combination, the experimental reflectance is compared to the simulated reflectance obtained from MC simulations ($R_{\text{sim}}$). To do so, three Biomimic Optical Phantoms^28^ are acquired with the following properties: phantom A (λ=560  nm, $\mu_{a}=0.0566\;{\mathrm{mm}}^{-1}$, $\mu_{s}^{\prime }=1.17\;{\mathrm{mm}}^{-1}$), phantom B (λ=633  nm, $\mu_{a}=0.0066\;{\mathrm{mm}}^{-1}$, $\mu_{s}^{\prime }=0.491\;{\mathrm{mm}}^{-1}$), and phantom C (λ=560  nm, $\mu_{a}=0.115\;{\mathrm{mm}}^{-1}$, $\mu_{s}^{\prime }=1.98\;{\mathrm{mm}}^{-1}$). An immersion liquid (coconut oil) is placed on the phantoms’ surface to reduce the specular reflection and improve the reproducibility of the measurements, and it is included in the MC simulation.

**Fig. 11:**
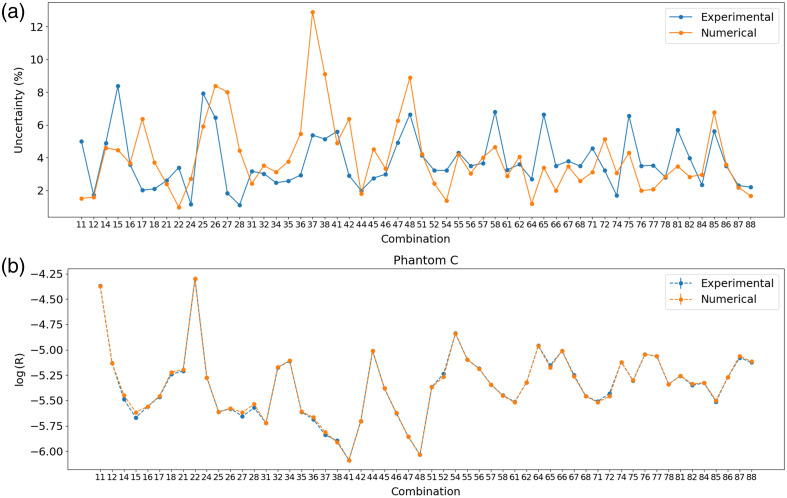
(a) Numerical and experimental uncertainty for each combination of illumination and detection fibers and (b) simulated and experimental reflectance values at λ=560  nm for phantom C.

The phantom’s subdiffusive parameter γ is not characterized by the manufacturer. To simultaneously evaluate γ of each phantom p and the calibration coefficient α of each fiber combination c, the following cost function is minimized:

$$\alpha^{c},\gamma^{p}=\underset{\alpha^{c},\gamma^{p}}{\min}\;\overset{n_{p}}{\underset{p=1}{\sum }}\overset{n_{c}}{\underset{c=1}{\sum }}|\frac{R_{\text{sim}}^{c,p}(\gamma^{p})-\alpha^{c}R_{\exp}^{c}}{R_{\text{sim}}^{c,p}(\gamma^{p})}\cdot \frac{1}{w_{\mathrm{tot}}^{c}}|$$ (4)

where $w_{\mathrm{tot}}^{c}=w_{\exp}^{c}+w_{\mathrm{num}}^{c}$ is the sum of the experimental and numerical uncertainties for each fiber combination, $n_{p}$ is the number of optical phantoms used to perform the fit, and $n_{c}$ the number of fiber combinations. The experimental uncertainty is evaluated by the standard deviation of ten repeated measurements on each phantom. The numerical uncertainty is evaluated by the MC simulation’s standard deviation, which is given by

$$S=\sqrt{\frac{1}{N_{\mathrm{col}}}\overset{N_{\mathrm{col}}}{\underset{i=1}{\sum }}(R_{i}-\overset{¯}{R}{)}^{2}}$$ (5)

where $N_{\mathrm{col}}$ is the number of collected photons, $R_{i}$ is the weight of each collected photon, and $\overset{¯}{R}$ is the reflectance value estimated by the MC simulation. Figure 11(a) shows the uncertainty of each fiber combination for both cases. It can be observed that the experimental uncertainty is higher for fibers with a higher tilt due to the stronger specular reflection, which makes the signal more sensitive to the probe positioning, whereas the numerical uncertainty is higher for fibers at larger SDS because fewer photons are collected. To reduce the effect of specular reflection, a thin layer of coconut oil is added to the surface of the phantom and included in the simulations in the form of a thin layer of one voxel thickness at the surface of the tissue. This oil is chosen because it is transparent in the visible spectrum, biocompatible, and has a refractive index (n=1.45) quite similar to optical fibers (1.43), phantoms (1.51), and biological tissues (1.51). Figure 11(b) shows the simulated and experimental reflectance after the signal processing and calibration. A log scale is chosen for visualization because the signal difference between the fiber combinations is large. After estimating $\alpha^{c}$ and $\gamma_{p}$ from each fiber combination on the three optical phantoms using Eq. (4), mean misfit values on the reflectance of 6.7%, 7.2%, and 2.0% and γ values of 1.3, 1.5, and 1.1 are obtained for the phantoms A, B, and C, respectively.

### 4.3 Validation on Bilayer Optical Phantoms

**Fig. 12:**
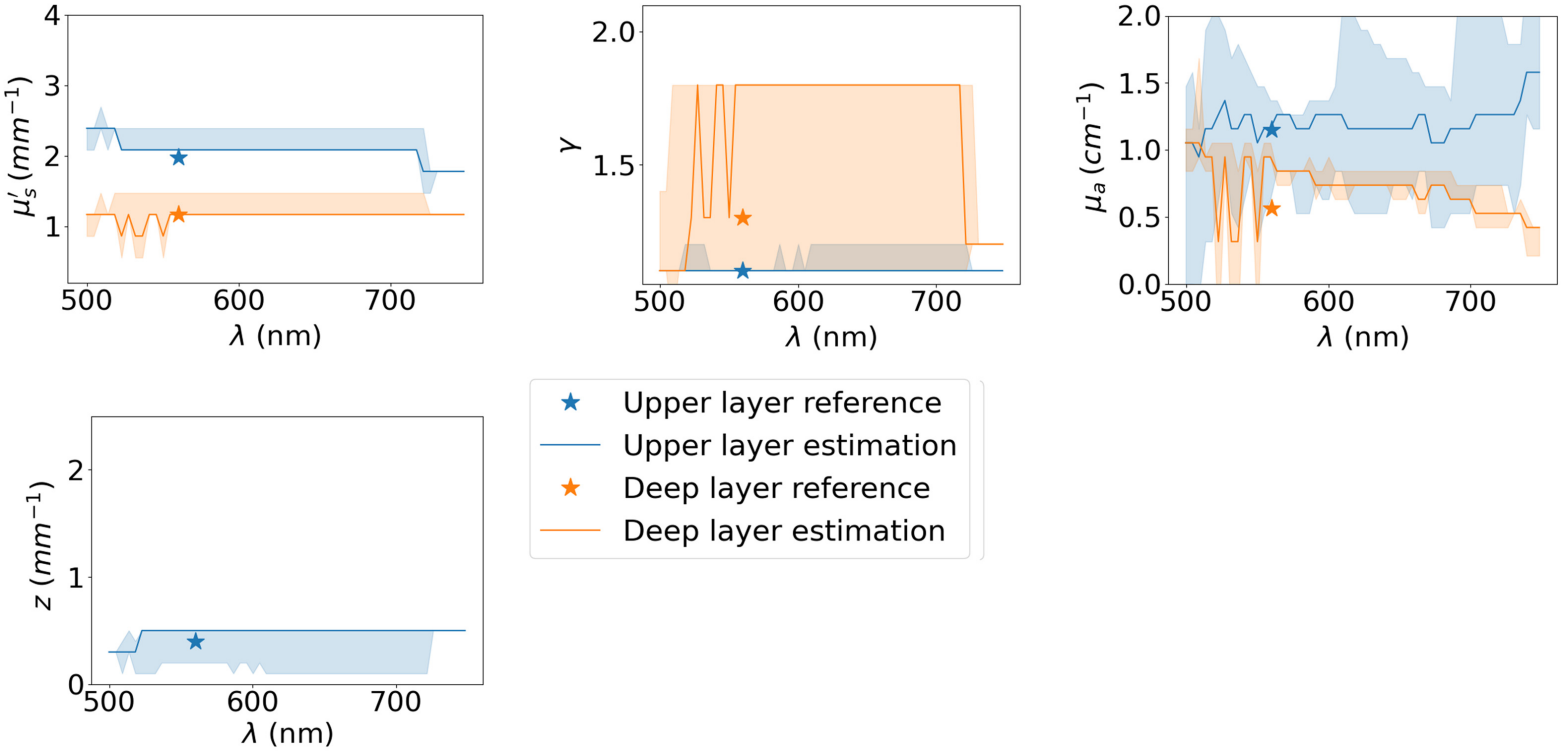
Spectral estimation of the IOPs of the bilayer phantom AC with a surface thickness of z=0.4  mm for the surface. IOPs of the upper layer are shown in blue and the deep layer in orange. The reference points from the phantom manufacturer are represented by the star symbol. The sudden spectral variations in the IOP estimation are due to the coarse discretization of the LUT.

Phantom C is fabricated to obtain seven thin slices of 0.1, 0.2, 0.3, 0.4, 0.5, 1, and 1.5 mm that are placed on the surface of phantoms A and B, labeled AC and BC, respectively, to create 14 bilayer phantoms. Coconut oil is added to the surface of both layers to reduce specular reflection due to the air interface. Phantom BC has a strong contrast between both layers, whereas AC has a weaker one. Figure 12 shows an example of the spectral IOP estimation for phantom AC with an upper layer thickness of z=0.4  mm as well as the reference values provided by the phantom manufacturer. The estimation error bars are obtained by propagating the total uncertainty presented in Fig. 11(a) into the inverse problem, i.e., by solving the inverse problem successively with $R_{\exp\pm }=(1\pm w_{\mathrm{tot}})R_{\exp}$, where $w_{\mathrm{tot}}$ is the total uncertainty of each fiber combination. We see that all other IOPs are properly estimated, but the uncertainty of estimation is large for the $\mu_{a1}$ (in the upper layer) and $\gamma_{2}$ (in the deep layer). The large uncertainty on $\mu_{a1}$ is explained by the relatively short path length within the upper layer because of its thinness. The large uncertainty on $\gamma_{2}$ seems to be related to the coarse discretization of this parameter in the LUT, which also explains the observed unstable spectral estimation of this property. The coarse discretization of the LUT leading to a large uncertainty of certain IOPs is the main limitation of the simultaneous estimation model. Indeed, each axis of the LUT is discretized by only 10 points because of the high computation cost associated with LUT generation.

**Fig. 13:**
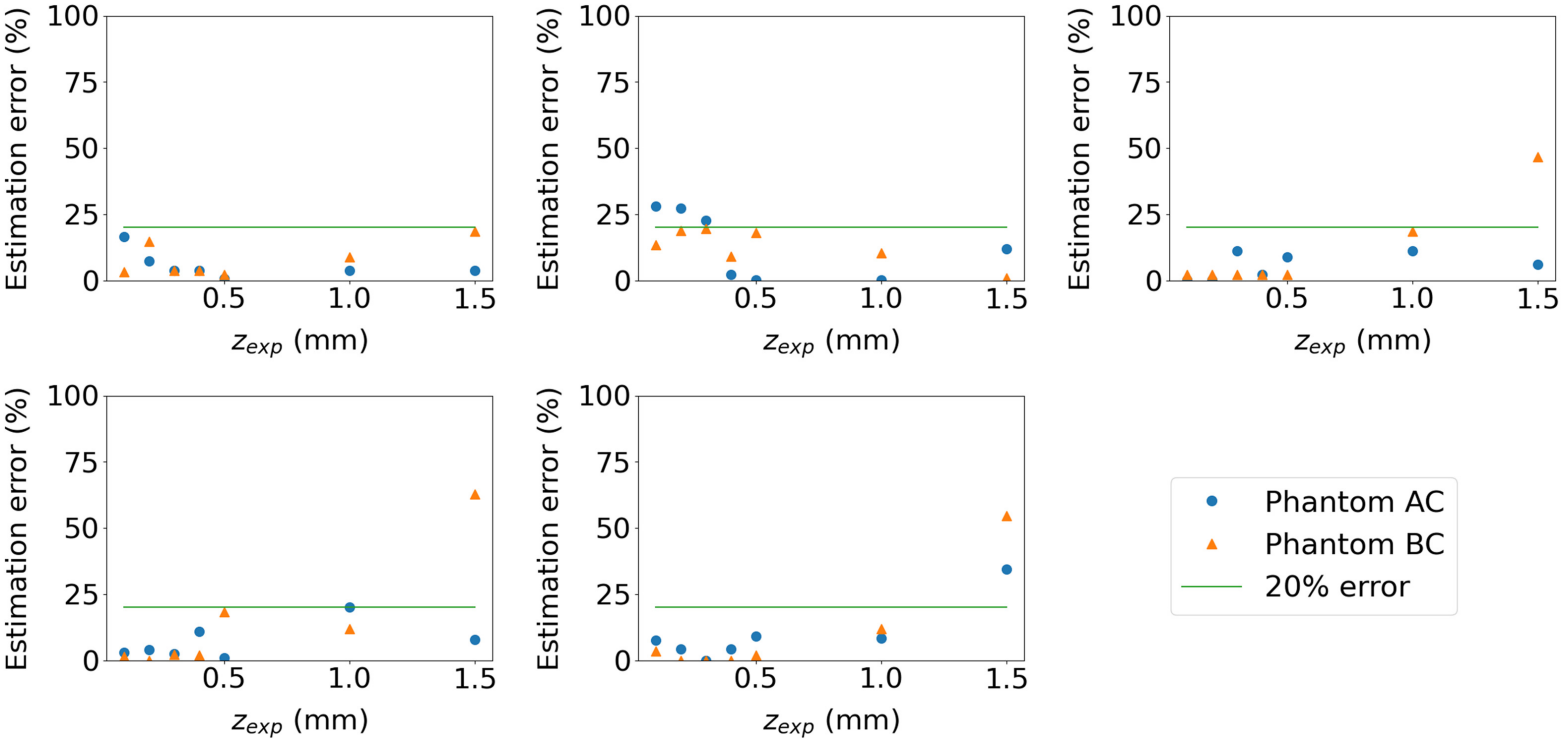
IOP estimation error at the reference wavelength for phantoms AC and BC as a function of the upper layer thickness.

The process of measuring the bilayer phantom and estimating the spectral IOPs is repeated for each of the 14 bilayer phantoms. In Fig. 13, the estimation error is reported as a function of phantom upper layer thickness where the superficial layer represents phantom C and where the deep layer represents either phantom B or A. The reference values used for the IOPs are those given by the phantom manufacturer at 560 nm. We see that the estimation of the upper layer thickness degrades for $z_{\exp}=1.5\;\mathrm{mm}$. This is because, as seen in Fig. 8(d), the photons collected by the probe mainly interact with a region shallower than 1.1 mm. Furthermore, the estimation of the upper layer’s absorption coefficient degrades when this layer thickness is very small (0.1 to 0.2 mm) because of the short path length of the photons within this layer, which is a physical limit of DRS. Overall, the estimation error is less than 20%, except for the absorption coefficient in the low-contrast phantom AC for $z_{\exp}\leq 0.1\;\mathrm{mm}$ and the IOPs of the deep layer of both phantoms when $z_{\exp}=1.5\;\mathrm{mm}$. A relative error of 20% seems to be a reasonable goal for bilayer tissue estimation.^5^^,^^6^^,^^8^ These results are very promising and are, to our knowledge, the first reported case of quantitative IOP estimation in bilayer media within the subdiffusive regime.

## 5 Conclusion

Part 1 of this paper proposed a methodology for the design of new compact probes that can quantitatively estimate the values of the optical coefficients in a localized manner. Part 2, taking advantage of this methodology, focuses on the design, characterization, realization, and experimental validation of the novel srDRS probe, which allows quantitative estimation of optical properties in bilayer tissues within the subdiffusive regime. Specifically, through numerical analysis, it was observed that fibers at short SDS are essential for accurate estimation of the scattering properties while fibers with larger SDS are essential for estimation of the absorption coefficient. By sampling a wide variety of SDS and tilt angles, the collected photons sample more thoroughly the diffusive medium’s scattering phase function and also lead to a more varied distribution of photon path lengths within the tissue, which contributes to a more compact cost function. A more compact cost function improves the robustness of the IOP estimation, which is critical for characterizing bilayer tissues due to the larger number of unknown parameters. Using synthetic data, the probe was shown to theoretically be effective for differentiating melanocytic nevi from melanoma. However, this capacity depends on the SNR of the measurements, the melanoma depth, and the optical contrast between the melanoma and its surrounding medium, which have each been characterized. These limits could be modified using a different geometry, for example by working with larger SDSs or with fibers tilted in opposite directions. This was not suitable for the considered application of early melanoma detection because of their limited size. Based on this numerical study, a custom probe for early melanoma detection was designed, built, and integrated into a typical DRS setup. A signal processing algorithm and a probe calibration scheme were presented, which resulted in a good fit between the simulated and experimental reflectance values in homogeneous phantoms. Probe performance, assessed by calculating spectral IOP estimates to 14 different well-characterized bilayer phantoms, exhibits an estimation error of less than 20%. The only cases where the error was larger than 20% are for the estimation of absorption coefficient in a low-contrast phantom with a very thin upper layer (≤0.2  mm) and for the IOPs of the deep layer when the upper layer thickness is larger than 1.5 mm.

The main limitation of the proposed model is the coarse discretization of the IOPs due to the high computational cost of generating a 7D LUT. To generate this large grid, a GPU-based MC simulation program was used in conjunction with state-of-the-art graphics cards and the Digital Research Alliance of Canada’s computing clusters to parallelize the problem across 100 computing nodes. To further improve discretization, a possible approach is to use more powerful computational tools, which may, for example, be brought by quantum computing. Another interesting improvement to the project would be to use machine learning to further optimize the probe’s geometry. The next logical step of this project is to validate the probe’s ability to characterize bilayer diffusive media directly *in vivo* and to validate the IOP estimation results with traditional biopsy methods. For *in vivo* measurements, careful attention should be paid to probe pressure as it is reported to affect IOP estimation.^32^^,^^33^

## 6 Appendix: Impact of Tilt Angle on Cost Function Shape and Optical Microscopy Probe Tip

**Fig. 14:**
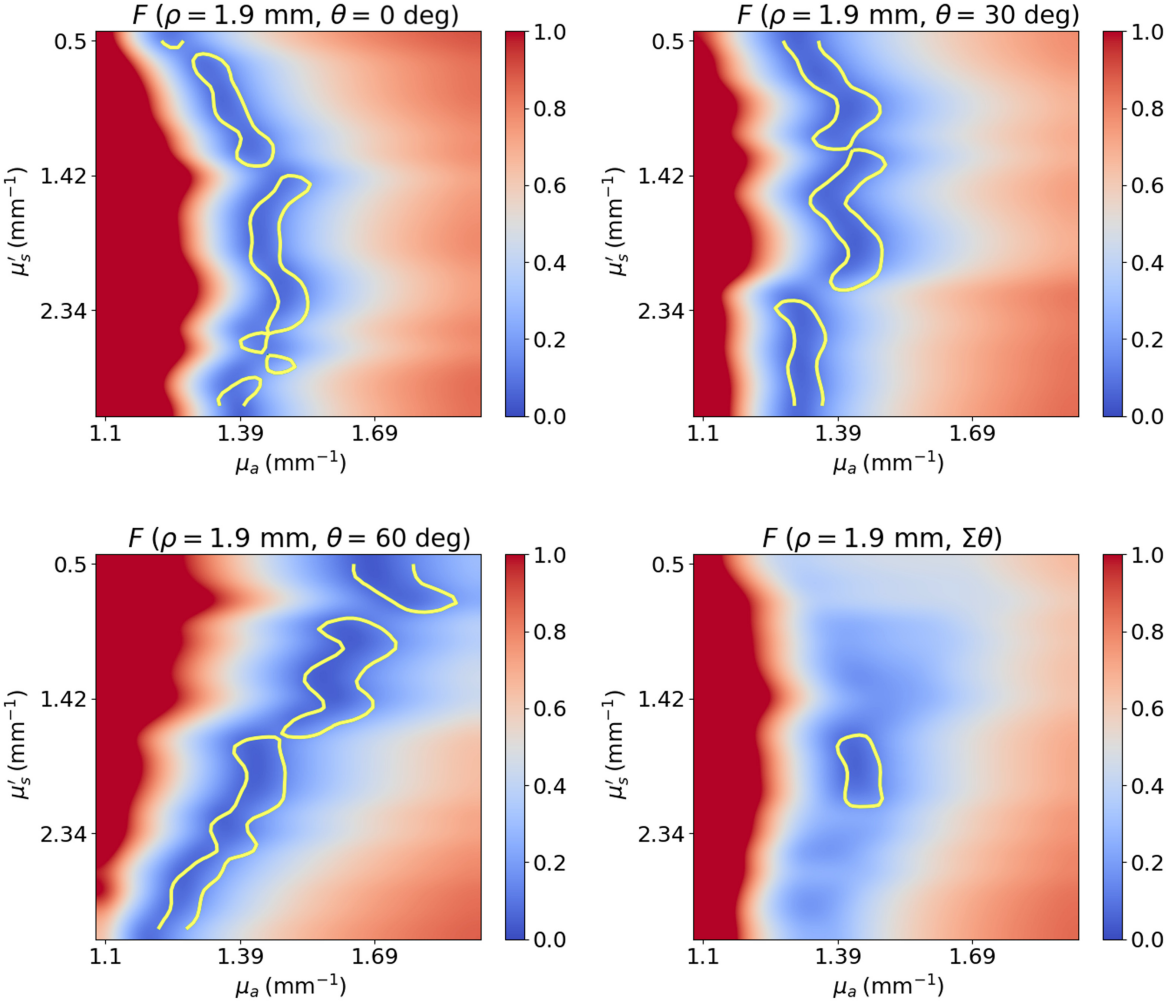
Cost function in the $\left(\mu_{s}^{\prime },\mu_{a}\right)$ plane for fibers at an SDS of 1.9 mm for tilt angles of 0 deg, 30 deg, and 60 deg and for the average of the three fibers. The yellow contour line delimits the region where the value of the cost function is less than 10%.

Figure 14 shows an additional example of the cost function shape as a function of tilt angle, this type in the plane of the reduced scattering coefficient and the absorption coefficient. Similarly, it can be observed that by varying the tilt angle, the shape of the cost function is modified, which yields to a convex and well-defined cost function when averaging the information from the three different tilt angles.

**Fig. 15:**
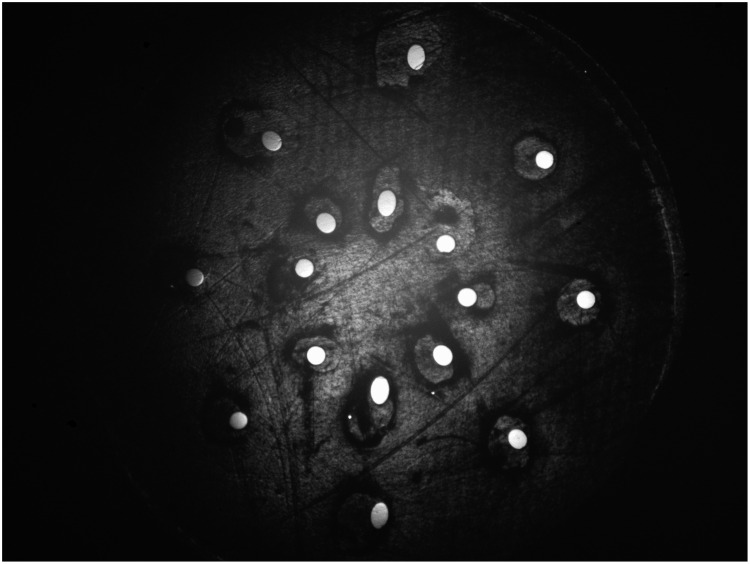
Optical microscopy of the probe’s tip to evaluate the exact positioning of the fibers.

Figure 15 shows the image of the probe's tip acquired by optical microscopy. The exact position of the fibers were determined by analyzing this image and these positions were incorporated in the Monte Carlo simulations.

## Data Availability

Data and code developed in this study are available upon reasonable request to the corresponding author.
